# Inter-Professional Collaboration and Patient Mortality: Protocol for a Systematic Review and Meta-Analysis

**DOI:** 10.3390/nursrep10010003

**Published:** 2020-09-08

**Authors:** Sandesh Pantha, Martin Jones, Richard Gray

**Affiliations:** 1 School of Nursing and Midwifery, La Trobe University Bundoora, Melbourne, VIC 3083, Australia; r.gray@latrobe.edu.au; 2 Department of Rural Health, University of South Australia, Whyalla Norrie, SA 5608, Australia; Martin.Jones@unisa.edu.au

**Keywords:** inter-professional collaboration, communication, patient outcomes, systematic review

## Abstract

Inter-professional collaboration is a process in which health professionals from different disciplines work together, sharing their ideas and opinions to plan evidence-based care. Nurses and doctors spend most of their time providing direct patient care. Therefore, effective interprofessional collaboration may be important in ensuring safe and effective patient care. There are no systematic reviews that have evaluated the association between nurse–doctor collaboration and patient outcomes in medical and surgical settings. We will conduct a systematic search of five key databases MEDLINE, EMBASE, PsycInfo, CINAHL, and the Cochrane register. We will include observational and experimental research that tests the association between levels of inter-professional collaboration and medical and surgical inpatient mortality. Two reviewers will independently conduct title and abstract, full-text screening, and data extraction. The Effective Public Health Practice (EPHPP) tool will be used to determine the quality of the included studies. If sufficient studies are available, we will undertake a meta-analysis. The protocol is registered with the international prospective register of systematic reviews (PROSPERO-CRD42019133543).

## 1. Introduction

Inter-professional collaboration is a process in which health professionals from different disciplines work together sharing their ideas and opinions to plan high quality, evidence-based care to ensure optimal patient outcomes [1,2]. Joint ownership of shared decisions is a key element for collaboration [3]. The Canadian inter-professional competency frameworks conceptualized inter-professional collaboration as a fusion of six domains-clarity of roles, patient-centered care, team dynamics, leadership, communication, and resolution of internal conflicts [4]. Xyrichis, Reeves, and Zwarenstein have illustrated inter-professional collaboration as a range of activities, including communication, discussion, teamwork, education, and networking among health care professionals [5,6].

### 1.1. Why Is Nurse–Doctor Interprofessional Collaboration Important?

Effective interprofessional collaboration between clinicians is important to ensure that patients receive optimal care and treatment, based on the best available evidence [7,8]. Authors have also suggested that improved interprofessional collaboration may reduce hospital length of stay [9], help prevent hospital readmissions [10], improve retention of clinical staff [11,12,13] staff, and enhance patient satisfaction with their care [12,13]. Health professionals from different backgrounds, including nurses, doctors, paramedics, and physiotherapists, bring a diverse set of skills to clinical practice [14,15,16]. Nurses and doctors spend a considerable amount of time providing direct patient care [17,18,19,20]. For example, Wenger et al. shadowed 36 internal medicine residents in practice at a University teaching hospital for a total of 700 h [17]. The authors reported that residents spent almost 90% of their time in direct and indirect patient care. A study of 767 nurses from 36 hospitals in the United States of America reported nurses spent two-thirds of their time in patient-related activities [18]. Nurses and doctors have different scopes of practice about the patient care they provide [7,8]. Consequently, effective collaborative working may influence clinical outcomes for hospitalized patients.

Mortality is one of the most important, accurately reported [21], and widely accepted patient outcomes. It is also a generally accepted indicator for measuring hospital performance and disease surveillance [22,23]. Death during or immediately after an inpatient admission could depend upon patient-related factors such as age, number of co-morbidities, the severity of underlying conditions, and the complexity of procedural interventions [24,25]. Other causes of patient death, which may be preventable, include medication errors [26,27,28], failure of recognition of severe adverse events [27], delay in diagnosis, and treatment initiation [7]. These preventable deaths may be influenced by hospital-related factors rather than patient characteristics [29]. Effective nurse–doctor working could prevent some of these preventable deaths and contribute to reducing mortality rates. For example, in a before and after study involving 423 nurses, the implementation of an enhanced communication protocol resulted in a reduction in mortality due to severe adverse events [27]. 

Readmission is an alternative measure of hospital performance. There is an ongoing debate about using readmission as a valid measure [30]. However, readmission can be difficult to measure because patients may be readmitted to a different hospital [31]. Yermilov et al. analyzed hospital readmissions among 2023 pancreatic cancer patients who underwent pancreaticoduodenectomy. Out of 1194 patients readmitted within one year of operation, almost half (47%) were hospitalized in a different hospital [32].

### 1.2. Why Is It Important to Do This Review?

Three systematic reviews have evaluated the effect of multidisciplinary team care interventions on patient outcomes. For example, Pannick et al. conducted a systematic review of 30 studies involving 66,548 patients admitted to general medical wards. In their meta-analysis, the authors reported that collaborative team care interventions were not effective at reducing in-hospital mortality (relative risk = 0.93, 95% CI = 0.82, 1.05) [11]. Reeves et al. reported a systematic review of interventions to enhance interprofessional teamwork [12]. The review included nine trials involving 5540 patients. The authors report that interventions—for example, checklists and bedside ward rounds—led to improved patient and clinician satisfaction. Martin et al. reported a systematic review of the nurse–doctor interprofessional team working on patient outcomes in primary care, which included 14 randomized control trials involving 5530 patients. The authors reported that, in the long run, nurse–doctor interprofessional team working could positively influence the physical, emotional, and functional wellbeing of patients [10]. In summary, these reviews suggest that good interprofessional team working can positively impact patient outcomes. None of these systematic reviews measured the amount of interprofessional collaboration among team members. The amount of collaboration may be equally important for enhancing patient outcomes. The association between levels (or amount) of interprofessional collaboration and patient outcomes has not previously been the subject of a systematic review. It is, therefore, important to understand the association between nurse–doctor collaboration and patient outcomes.

### 1.3. Review Question

Is there an association between the amount of nurse–doctor interprofessional collaboration and medical or surgical inpatient mortality?

## 2. Methods

This protocol complies with the guidelines of the Preferred Reporting Items for Systematic Review and Meta-Analysis Protocols (PRISMA-P) 2015 statement (Supplementary material 1). The review was prospectively registered with the International Prospective Register of Systematic Reviews (PROSPERO) (CRD42019133543).

### 2.1. Eligibility Criteria

Studies will be included based on the following criteria:

- Experimental (example, randomized controlled trials) and observational (cohort, case-control, or cross-sectional) studies.
- The exposure is nurse–doctor interprofessional working (collaboration, communication, discussion, teamwork, education, and networking) [5,6] measured by using validated tools, checklists, scales, or instruments.
- Patients are adults aged 18 years or over.
- Fieldwork was conducted in general or specialist medical or surgical inpatient wards.
- The outcome is any measure of mortality that may include deaths whilst in the hospital or 30-days following discharge. We will include deaths within 30 days of discharge from the hospital as there is evidence that early deaths, preferably within a month of hospital admission could be influenced by the quality of care received during a hospital stay [29,33].
- Studies conducted in emergency departments or inpatient psychiatric units will be excluded as they are different clinical settings that would likely introduce considerable heterogeneity.
- Published in English.

### 2.2. Information Source

Five online databases—the Medical Literature Analysis and Retrieval System Online (MEDLINE), Excerpta Medica (EMBASE), PsycInfo, Cumulative Index to Nursing and Allied Health Literature (CINAHL), and the library of the Cochrane Collaboration—will be searched using a combination of keywords and Medical Subject Heading (MeSH) terms. MEDLINE, EMBASE, and PsycInfo will be accessed via Ovid. The EBSCOhost and Wiley platforms will be used to access CINAHL and the Cochrane library, respectively. There will be no restriction for date or language in the search strategy.

We will not search for grey literature because studies may not have been through an exhaustive peer-review process [34].

We will not search for the references of included studies because this introduces considerable (observation and selection) bias into the review methodology [35].

### 2.3. Search Strategy

To develop the search strategy, we disaggregated our research question into three discreet concepts: Inter-professional collaboration, healthcare professionals (nurses and doctors), and mortality. The Peer Review of Electronic Search Strategies (PRESS) suggests developing a search strategy using a combination of indexing terms such as medical subject headings (MeSH) and text words (full and various truncations) that can then be combined using the bullion operators (AND, OR) [36].

The search strategy was developed initially in MEDLINE (Supplementary material 2) and then customized for other databases. We developed our search strategy in collaboration with a University information scientist. The search strategy was reviewed by a second information scientist. Feedback from the review was incorporated into the final search strategy. For example, the search term “intersectoral” was added. 

Our search strategy in MEDLINE yielded more than 8000 citations. We, therefore, decided, again in consultation with an information scientist, to restrict our search to title and abstract only.

### 2.4. Data Management

The output from each database will be exported (as a .enl file) to Endnote X9.2 (Clarivate Analytics, Philadelphia, PA, USA). We will use Covidence, an online software package, to undertake title and abstract and full-text screening. Covidence is an established package for managing systematic reviews [37,38]. References from the endnote files will be imported to Covidence (as a .xml file). Any duplicates will be identified by Covidence and the multiples of the citation removed.

The number of papers at each stage of the review will be recorded and reported in a PRISMA flowchart [39]. We will report the date when an individual database was searched.

## 3. Screening and Selection of Studies

Study selection will follow a two-step process: 1. title and abstract screening and 2. full-text screening. At each stage, two reviewers will screen studies against predefined inclusion and exclusion criteria. Any discrepancies between reviewers will be resolved by a third member of the review team.

## 4. Assessment of the Methodological Quality (Risk of Bias) of Included Studies

We will use the Effective Public Health Practice Project (EPHPP) measure to assess the methodological quality of the included studies. The EPHPP tool has good psychometric properties and can be used across experimental and observational (cohort, case-control) studies [40]. Higher inter-rater agreement of the EPHPP tool compared to version 1.0 of the Cochrane collaboration risk of bias tool has been reported [41].

The EPHPP tool (https://link.springer.com/content/pdf/bbm%3A978-3-319-17284-2/1.pdf) has eight sections, each with between two and four items: 1. selection bias (two items), 2. study design (one item), 3. confounders (two items), 4. blinding (two items), 5. data collection tools (two items), 6. withdrawals and drop-outs (two items), 7. intervention strategy (three items), and 8. analysis (four items). Each section is rated “strong”, “moderate” or “weak”. The final overall risk of bias is determined based on the number of strong and weak ratings in the first six items. Studies are rated: 1. strong “no weak ratings and at least four strong ratings”, 2. moderate “less than four strong ratings and one weak rating”, and 3. weak “two or more items are rated weak” [40]. We will report the findings of the risk of bias against the six core items in a table.

We will check and report if ethical approval was obtained for the individual studies.

It is important to check that systematic reviews do not include any studies that have been retracted [42,43,44]. We will check the entry on the journal website to determine if the paper has been retracted or if there are any expressions of concern. We will document the date that this check was undertaken.

## 5. Data Extraction and Management

Two reviewers will independently extract data from eligible studies, using a data extraction form designed by the review authors. We will pilot test the tool on two included studies and make any necessary refinements based on reviewer feedback. We will extract the following information from the included studies:

1. Citation (surname and initial of first author, title, year of publication).
2. Study design (coded, randomized control trial, cohort, case-control, survey, other).
3. Study setting (coded, medical, surgical, both).
4. The country where fieldwork was undertaken.
5. Period of data collection.
6. Sampling strategy.
7. Participants (type of clinician and number).
8. The measure of interprofessional collaboration (mean and standard deviation).
9. A measure of mortality (outcome reports as Risk Ratio or Odds Ratio).

If necessary, we will email the corresponding author for any essential information about the included study not reported in the manuscript. We will make a note of our attempt to contact authors to obtain additional information/clarification about included studies.

If multiple studies are produced by using the same data set, we will extract the data from the individual studies and then coalesce information across the studies [45].

Data extracted from the included studies will be reported in a summary table.

### Test of Heterogeneity

We anticipate a degree of heterogeneity between included studies. Heterogeneity will be determined using the *I^2^* test. We will apply a standard threshold to determine heterogeneity (25% “low”, 50% “moderate” and 75 “high”). The *I*^2^ test will be used because heterogeneity can be calculated even when studies use different measures of association (e.g., odds ratio or relative risk) [46].

## 6. Meta-Analysis

A meta-analysis will be used to pool findings from included studies (p11) [47] using the Review Manager software package (RevMan, Version 5.3, Cochrane collaboration, London, UK). We will undertake a meta-analysis if more than two included studies report mortality as an outcome of interest. We will use a random-effects model if the observed heterogeneity among the included studies is moderate to high (i.e., I^2^ ≥ 25%).

We will present a forest plot of the pooled estimates as a risk ratio (95% confidence intervals). Where individual studies report an Odds Ratio as a measure of association, we will convert it to Risk Ratio using the procedures in RevMan Version 5.3.

### 6.1. Sensitivity Analysis

Sensitivity analysis allows us to check if the results from a meta-analysis were affected by the inclusion of certain studies based on predefined criteria. We will undertake a sensitivity analysis to determine if the outcome is influenced by omitting one or more studies from the analysis. For example, we will conduct a sensitivity analysis to examine the effect of bias by removing studies at high risk.

### 6.2. Subgroup Analysis

Subgroup analysis will help us to understand the mean effect and variation in different study populations. For example, we will consider a subgroup analysis to determine if there is any difference in outcomes between medical and surgical wards. We will calculate the mean effect and variance for each group and compare between subgroups. Subgroup analysis will only be considered if there are at least five studies in each group [47].

### 6.3. Meta-Biases

We will conduct a funnel plot analysis to identify publication bias among the studies included in the review. However, it has been suggested that the sensitivity of a funnel plot analysis to detect publication bias is limited if the meta-analysis is conducted with less than ten studies [48,49].

## 7. Discussion

This systematic review and meta-analysis aim to synthesize evidence of the association between nurse–doctor interprofessional collaboration and mortality among medical and surgical inpatients. We found no systematic reviews that have examined the association between interprofessional collaboration (the amount of) and patient outcomes. This systematic review and meta-analysis will help us understand the gap in the existing literature about how nurse–doctor interprofessional collaboration influences the patient outcome. The outcomes from our systematic review will inform interprofessional working in the medium term to benefit the health and wellbeing of people that use health services.

## Supplementary Materials

The following are available online at https://www.mdpi.com/2039-4403/10/1/3/s1, Supplementary material 1: PRISMA-P Checklist, Supplementary material 2: MEDLINE search strategy.
