# Supplementary material for: Inter-Professional Collaboration and Patient Mortality: Protocol for a Systematic Review and Meta-Analysis
Source: Nurs Rep. 2020 Sep 8;10(1):15–22. doi: 10.3390/nursrep10010003 (PMC8608100; doi:10.3390/nursrep10010003)
Supplement: Supplementary file 1 [file nursrep-10-00003-s001.zip › Supplementary material 2_MEDLINE search strategy.pdf]

Appendix 2: MEDLINE Search strategy:

1. Patient Care Team/ or intersectoral collaboration/ or interprofessional relations/ or interdisciplinary communication/ or Physician-Nurse Relations/
2. ((Interprofessional or "Inter professional" or Intersectoral or interdisciplinary or "inter disciplinary") adj3 (collaborat* or co-operat* or cooperat* or communicat* or interact* or relation* or discussion* or round* or meeting or teamwork or care)).ti,ab.
3. ((Multiprofessional or "multi professional" or multidisciplinary or "multi disciplinary") adj3 (collaborat* or co-operat* or cooperat* or communicat* or interact* or relation* or discussion* or round* or meeting or teamwork or care)).ti,ab.
4. ((crossdisciplinary or "cross disciplinary") adj3 (collaborat* or co-operat* or cooperat* or communicat* or interact* or relation* or discussion* or round* or meeting or teamwork or care)).ti,ab.
5. ((transdisciplinary or "trans disciplinary") adj3 (collaborat* or co-operat* or cooperat* or communicat* or interact* or relation* or discussion* or round* or meeting or teamwork or care)).ti,ab.
6. ((Interoccupation* or "inter occupation" or "inter occupational") adj3 (collaborat* or co-operat* or cooperat* or communicat* or interact* or relation* or discussion* or round* or meeting or teamwork or care)).ti,ab.
7. (("doctor nurse" or "physician nurse") adj3 (collaborat* or co-operat* or cooperat* or communicat* or interact* or relation* or discussion* or round* or meeting or teamwork or care)).ti,ab.
8. 1 or 2 or 3 or 4 or 5 or 6 or 7
9. Mortality/ or death/ or hospital mortality/
10. (death* or mortalit* or fatalit*).ti,ab.
11. 9 or 10
12. 8 and 11
